# Assessment of expertise in morphological identification of mosquito species (Diptera, Culicidae) using photomicrographs[Fn FN1]

**DOI:** 10.1051/parasite/2022045

**Published:** 2022-10-06

**Authors:** Nil Rahola, Filiz Günay, Murat Öztürk, Bulent Alten, Hanan A. Aqeehal, Walid K. Saadawi, Taher Shaibi, Mihaela Kavran, Dušan Petrić, Jelena Mitrović, Igor Pajovic, Enkelejda Velo, Përparim Kadriaj, Elton Rogozi, Viola Jani, Arsen Manucharyan, Lusine Paronyan, Samer Sawalha, Youmna M’ghirbi, Ali Bouattour, Adel Rhim, Ahmed Ouni, Abdallah M. Samy, Shaimaa Abozeid, M’hammed Sarih, Najlaa Assaid, Soukaina Arich, Nikolina Sokolovska, Elizabeta Janceska, Kamal Eddine Benallal, Nabil Haddad, Renée Zakhia, Nesade Muja-Bajraktari, Kurtesh Sherifi, Majeda Arbaji, Jelena Marić, Violeta Santrac, Nato Dolidze, Philippe Boussès, Isra Deblauwe, Francis Schaffner, Vincent Robert

**Affiliations:** 1 MIVEGEC Unit, Montpellier Univ., IRD, CNRS Montpellier France; 2 Hacettepe Univ., Fac. of Science, Dept. of Biology, VERG Laboratories, Beytepe Ankara Turkey; 3 Head of the Research Laboratory of Parasitology and Vector Borne Diseases – National Centre for Disease Control, Gorji, near to Sports City Pox 71171 Tripoli Libya; 4 Head Vector Borne Diseases Control Department – National Centre for Disease Control, Gorji, near to Sports City Pox 71171 Tripoli Libya; 5 Zoology Department University of Tripoli, National Center for Diseases Control University Road. 1 13793 Tripoli Libya; 6 University of Novi Sad, Faculty of Agriculture, Laboratory for Medical and Veterinary Entomology Trg Dositeja Obradovica 8 Novi Sad Serbia; 7 Department of Ecology and Environmental Improvement “PUC City Sanitation” Trebevicka 16 11030 Belgrade Serbia; 8 Biotechnical Faculty – University of Montenegro, Biotechnical Faculty Mihaila Lalića 15 81000 Podgorica Montenegro; 9 Institute of Public Health, Dep. of Epidemiology and Control of Infectious Diseases, Vectors’ Control Unit Str. “Aleksander Moisiu”, No. 80 Tirana Albania; 10 Head of Laboratory of Episootology, Ectoparasitology and Entomology 37 Davit Malyan str. Yerevan 0060 Armenia; 11 Head of Zoonotic & Parasitic Diseases Epidemiology Dpt, NCDC, MoH 12 Heratsi street Yerevan 0025 Armenia; 12 Vector Control Unit, Environmental Health Department, Ministry of Health Ajnadeen st. Ramallah Palestine; 13 Laboratoire de Virus, Vecteurs et Hôtes (LR20IPT02), Institut Pasteur de Tunis, Université Tunis El Manar, Tunisia 13 place Pasteur Tunis 1002 Tunisia; 14 Entomology Department, Faculty of Science, Ain Shams University, Abbassia Cairo 11566 Egypt; 15 Laboratoire des Maladies Vectorielles. Institut Pasteur du Maroc 1 Place Louis Pasteur 20 360 Casablanca Morocco; 16 Service de Parasitologie et des Maladies Vectorielles, Institut Pasteur du Maroc 1 Place Louis Pasteur 20 360 Casablanca Morocco; 17 Department for Vector and Pest Control with Laboratory of Entomology, PHI Center for public health-Skopje Blvd. 3rd Macedonian Brigade 18 1000 Skopje North Macedonia; 18 Laboratory for Virology and Molecular Diagnostics, Institute of Public Health, Health of Rep. of North Macedonia Skopje Republic of North Macedonia; 19 Laboratoire d’Eco-épidémiologie Parasitaire et Génétique des Populations, Institut Pasteur d’Algérie Route du Petit Staouéli Dely Ibrahim Alger Algérie; 20 Laboratory of Immunology and Vector-Borne Diseases, Faculty of Public Health, Lebanese University Street 37, Pierre Gemayel Campus Fanar-El Metn Lebanon; 21 University “Hasan Prishtina”, Prishtina, Republic of Kosovo. Faculty of Mathematics and Natural Sciences, Department of Biology Str. Mother Teresa. p.n. 10.000 Prishtinë Kossovo; 22 Faculty of Agriculture and Veterinary, University of Prishtina “Hasan Prishtina”, Prishtina, Kosovo, Boul. « Bill Clinton » , n.a. 10000 Prishtina Kosovo; 23 Parasitic and Zoonotic Diseases Division, National Malaria Control Program, Ministry of Health Amman Jordan; 24 Public institution Veterinary Institute of the Republic of Srpska “Dr. Vaso Butozan”, Center for Animal Health and Food Safety Branka Radicevica 18 Banja Luka 78000 Bosnia and Herzegovina; 25 Zoo Entomology Laboratory, R. Lugar Center for Public Health Research, National Center for Disease Control and Public Health, Georgia 99, Kakheti Highway 0198 Tbilisi Georgia; 26 Unit of Entomology, Department of Biomedical Sciences, Institute of Tropical Medicine Nationalestraat 155 2000 Antwerp Belgium; 27 Francis Schaffner Consultancy Lörracherstrasse 50 4125 Riehen Switzerland

**Keywords:** External Quality Assessment (EQA), Identification, Key, Vector, Gamification

## Abstract

Accurate identification of insect species is an indispensable and challenging requirement for every entomologist, particularly if the species is involved in disease outbreaks. The European MediLabSecure project designed an identification (ID) exercise available to any willing participant with the aim of assessing and improving knowledge in mosquito taxonomy. The exercise was based on high-definition photomicrographs of mosquitoes (26 adult females and 12 larvae) collected from the western Palaearctic. Sixty-five responses from Europe, North Africa and the Middle East were usable. The study demonstrated that the responders were better at identifying females (82% correct responses) than larvae (63%). When the responders reported that they were sure of the accuracy of their ID, the success rate of ID increased (92% for females and 88% for larvae). The top three tools used for ID were MosKeyTool (72% of responders), the ID key following Becker et al. [2010. Mosquitoes and their control, 2nd edn. Berlin: Springer] (38%), and the CD-ROM of Schaffner et al. [2001. Les moustiques d’Europe: logiciel d’identification et d’enseignement – The mosquitoes of Europe: an identification and training programme. Montpellier: IRD; EID] (32%), while other tools were used by less than 10% of responders. Responders reporting the identification of mosquitoes using the MosKeyTool were significantly better (80% correct responses) than non-MosKeyTool users (69%). Most responders (63%) used more than one ID tool. The feedback from responders in this study was positive, with the exercise being perceived as halfway between educational training and a fun quiz. It raised the importance of further expanding training in mosquito ID for better preparedness of mosquito surveillance and control programmes.

## Abstract (fr)

L’identification précise des espèces d’insectes est une exigence indispensable et difficile pour tout entomologiste, en particulier si l’espèce est impliquée dans des épidémies. Le projet européen MediLabSecure a conçu un exercice d’identification (ID) accessible à tout participant volontaire dans le but d’évaluer et d’améliorer les connaissances en taxonomie des moustiques. L’exercice était basé sur des photomicrographies haute définition de moustiques (26 femelles adultes et 12 larves) prélevées dans le Paléarctique occidental. Soixante-cinq réponses d’Europe, d’Afrique du Nord et du Moyen-Orient ont été utilisables. L’étude a démontré que les répondants étaient meilleurs pour identifier les femelles (82 % de réponses correctes) que les larves (63 %). Lorsque les répondants ont déclaré être sûrs de l’exactitude de leur ID, le taux de réussite de l’identification était meilleur (92 % pour les femelles et 88 % pour les larves). Les trois principaux outils utilisés pour les ID étaient MosKeyTool (72 % des répondants), la clé d’identification du livre de Becker et al. (38%) et le CD-ROM de Schaffner et al. (32 %), tandis que d’autres outils étaient utilisés par moins de 10 % des répondants. Les répondants déclarant identifier des moustiques à l’aide de MosKeyTool étaient significativement meilleurs (80 % de réponses correctes) que les non-utilisateurs de MosKeyTool (69 %). La plupart des répondants (63 %) ont utilisé plus d’un outil d’identification. Les commentaires des répondants de cette étude ont été positifs, l’exercice étant perçu comme à mi-chemin entre une formation pédagogique et un quiz amusant. Il a souligné l’importance d’étendre la formation complémentaire à l’identification des moustiques pour une meilleure préparation des programmes de surveillance et de contrôle des moustiques.

## Introduction

Vector-borne diseases are strongly affected by global changes such as modifications of ecosystems, climate change, changes in agricultural practices, increasing urbanisation, deforestation, and worldwide travel [7, 11, 12]. The European MediLabSecure project (https://www.medilabsecure.com) launched in 2014, aims to prevent vector-borne diseases by reinforcing an international network of laboratories and public health institutions in 22 countries to promote integrated surveillance of emerging arboviruses. These countries, which are in the Balkans, South Caucasus, the Middle East, North Africa and the Sahel by design, are not members of the European Union. Virology, entomology, and public health training sessions were made available to the laboratories on a regular basis soon after the network’s implementation to strengthen their theoretical competency and technical expertise.

Several training sessions in medical entomology focused on the persistent issue of species-level insect identification (ID), particularly with regard to mosquitoes (family Culicidae) that harbour numerous disease vectors with important implications for human and animal health. In fact, much like with medical diagnostics, the ID of specimens in entomology is a matter of utmost importance. Many approaches to ID are used, including morphological observation and molecular analysis that may ideally constitute two complementary approaches [9].

In February 2022, the MediLabSecure project launched an assessment of expertise in morphological ID of mosquito specimens among researchers in various countries. This assessment took the form of a fully open exercise to everyone who wanted to participate; they were member or non-member laboratories of MediLabSecure. The level of participation exceeded the organizers’ hopes, allowing us to analyse a unique dataset of 65 total usable responses. We think that this exercise provides lessons of a general scope and we report herein on the exercise itself based on photomicrograph examinations, but also the level of expertise of responders in terms of correct/incorrect responses, and the analysis of the various methods and processes used by the responders.

## Genesis of the exercise, materials and methods

This exercise is similar to an External Quality Assessment (EQA). The organizers that provided the quality statement were outside the laboratory’s organisation but they were not accredited according to any ISO standard. This is why the present EQA-like approach for assessing ID expertise is called “the exercise” in this article.

Initially, the organizers of the exercise planned to send a set of dead mosquitoes (adult females and larvae) by post or by transporter to be identified by MediLabSecure laboratory members in 16 European and Mediterranean countries. In the face of huge practical and regulatory obstacles, the organizers made an exercise relying on photographs, more precisely on one plate of photomicrographs per species, not restricted to the sole MediLabSecure framework.

**Figure 1:**
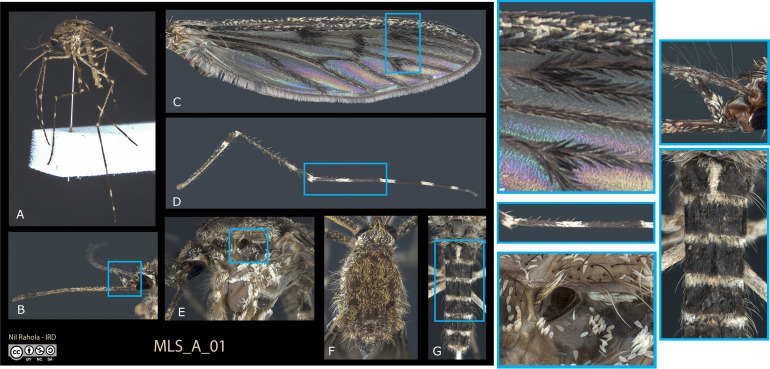
Example of a set of images used for the identification exercise: plate MLS_A_01 used for female identification (A) and magnification showing discriminating characters (boxed in blue) such as pale scales on maxillary palpus (B), wings spotted with dark scales (C), hind leg with a large median pale band on tarsomere 1 (D), presence of prespiracular setae (E), scutum without discriminating character (F) and apical half of abdominal terga without pale scales (G). This set of characters is typical of *Culiseta annulata*.

**Figure 2:**
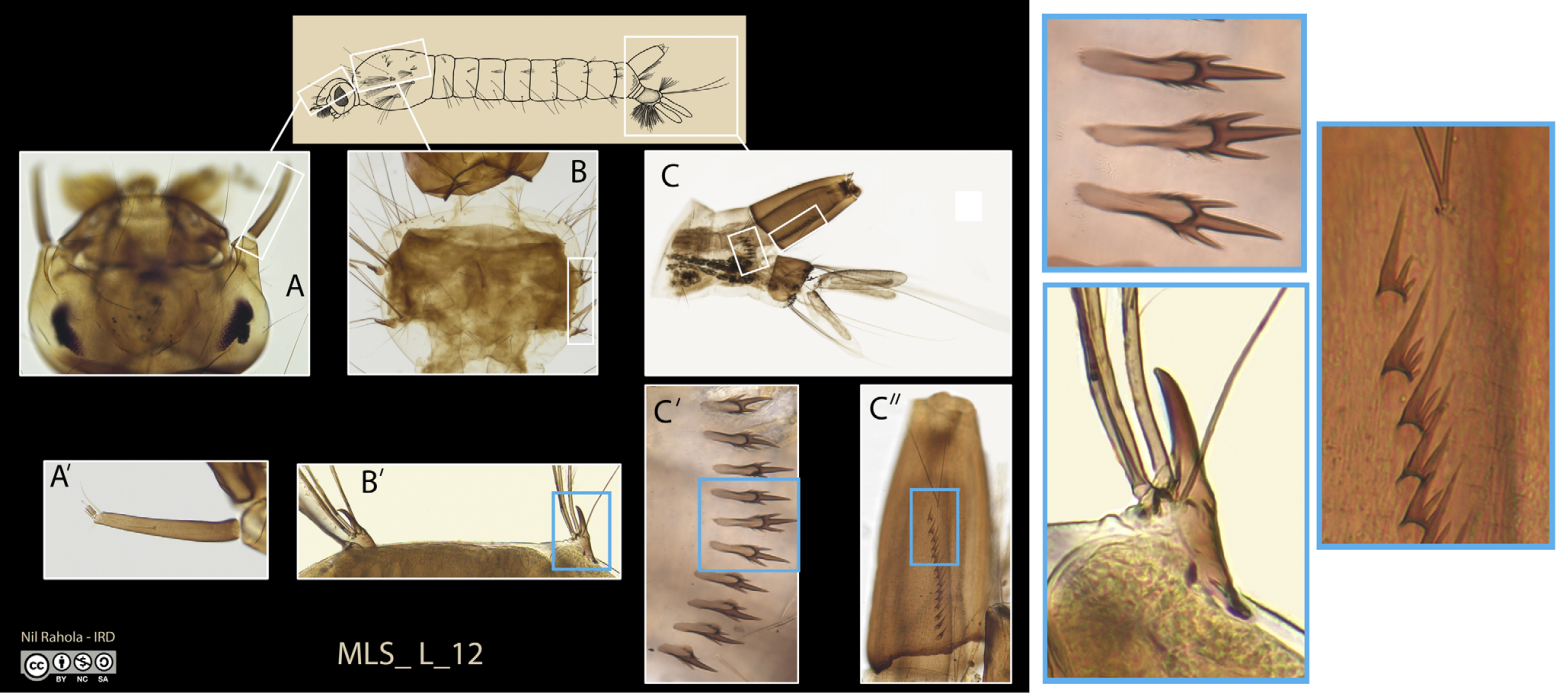
Example of a set of images used for the identification exercise: plate MLS_L_10 used for larva identification and magnification showing discriminating characters (boxed in blue) such as head (A) and antenna (A’), thorax (B) with meso and metathorax with long stout spine, pointed and hooked at the tip (B’), abdominal segments VIII-X (C) with comb composed of 10 teeth with a long median spine and strong smaller spines at the basis (C’) and pecten with 15 evenly spaced teeth (C’’). This set of characters is typical of *Aedes aegypti*.

Four characteristics were sought and verified for each photograph: high resolution imagery to allow large magnification of details, a significant depth of field, good contrast, and sound colour reproduction. In practice, in the MIVEGEC Laboratory at IRD, Montpellier, we used a Leica Z16ApoA stereomicroscope equipped with a DMC5400 camera. These materials were positioned on a vibration filtering table (CleanBench TMC M6) functioning on an air cushion (TMC’s Gimbal Piston Air Isolators). All pictures were made using a focus stacking technique (multiple images taken at different focus to extend the depth of field) within the LAS X software from Leica. All pictures were then processed in Adobe Photoshop 2021 to correct and adjust various parameters such as exposure, white balance and light curve. For female mosquitoes, the following features were taken into account when photographed: a general view of a specimen, a lateral view of the proboscis and head, a view of the right wing, a view of the left side of the hindleg, a lateral view of the thorax, and a dorsal view of the scutum and vertex, and a dorsal view of the abdominal terga (Fig. 1). A view of the anterior face of the foreleg was added for species of the genus *Orthopodomyia* owing to its significance in identifying this genus. For fourth instar larvae: a general habitus with indications of the parts photographed, a dorsal view of the head, a left lateral view of the terminal abdomen (segments VII–X), the comb, the siphon, the pecten teeth, an enlargement of the antenna (Culicinae only), an enlargement of palmate setae (Anophelinae only), and if needed, a dorsal view of the thorax, a ventral view of the mentum, and a dorsal view of abdominal segment V (Fig. 2).

The biological materials were females mounted on minuten pins and slide-mounted fourth instar larvae bleached before mounting on microscope slides, selected from the 144 species recorded in the Western Palaearctic, which encompasses the European and Mediterranean areas (145 species listed by [10] minus one homonym). These mosquitoes were obtained from the IRD Arthropodes d’Intérêt Médical collection ARIM (Arthropods of Medical Interest) in Montpellier, France. The specimens had been identified by specialists once they were included in the collection and additional expert confirmation was also performed for the exercise. Finally, the photomicrograph plates revealed 26 females and 12 larvae. A small number of plates were developed from a single specimen. Most of the time, the photographer required multiple specimens of the same species since some of them were damaged in some parts or were not representative of the character that was intended to be there. All the plates are available on the MIVEGEC website at https://mivegec.fr/fr/identiciels.

Preliminary control was performed with close colleagues to ensure that the exercise was feasible. Also, the fact that three responses were 100% correct (see below) constitutes a confirmation that the exercise was feasible.

Communications about this exercise were released in early February 2022 via several channels: the European and Mediterranean laboratories members of the MediLabSecure network, the network *Aedes* Invasive Mosquitoes-COST Action, VectorNet, MIVEGEC/IRD, the International Network of Institut Pasteur, the European Mosquito Control Association, the French mosquito control operators, the research unit ASTRE of CIRAD, the BioInsecte network, the Global Vector Hub, and the vector network of the French Agency for Food, Environmental and Occupational Health & Safety (ANSES).

The information on this exercise was associated with (i) a “read me first” file in PDF format (Supplementary files 1 and 2), (ii) a template to finalise the responses (genus and species were required; methods used, time spent in minutes, if the ID is thought to be sure or doubtful, were optional) in Microsoft Word™ format (Supplementary file 3), along with an e-mail address to return the IDs, and (iii) a Uniform Resource Locator (URL) to download the photomicrographs plates of 26 females and 12 larvae in JPG format. Participation in the exercise was free of charge and without subsequent obligation. The responder, a single person or a group of persons, was free to use (or not) the ID tool/tools of his/her/their choice. Also, the responders allowed the organizers to develop the global anonymized set of responses in some scientific reports and publications.

Responses were received between 9 February and 7 April 2022. Every responder received feedback on the accuracy of his/her/their IDs within seven days.

The responses were gathered in a single data sheet in Microsoft Excel™ 2016.

Statistical analysis was fundamental: distribution in a 2 × 2 contingency table was tested by the Fisher’s exact test and the comparison of two means by the Mann–Whitney *U* test, always two-tailed; and the degree of association between two quantitative variables was measured by a Pearson correlation coefficient *r*. The calculation was performed via BiostaTGV https://biostatgv.sentiweb.fr/. A few statistical tests were performed for several reasons, including the fact that (i) no random procedure was possible with such a design based on the free involvement of participants, (ii) the motivations of responders may vary between hard training and lazy fun, (iii) the groups are hardly comparable, especially between MediLabSecure responders (desired participation) vs. non-MediLabSecure (unsolicited participation), and (iv) the anonymity of the responders must be preserved.

## Results

In total, 66 responses were received, of which one was off-topic and eliminated, yielding a usable dataset of 65 responses.

### Respondents

Among the 65 responses, 24 (37%) came from MediLabSecure laboratories distributed across all 16 MediLabSecure countries of the Balkans, North Africa, the Middle East, Black Sea and South Caucasus. The geographic origin of the 65 responses may be organized into six groups: France, mainly belonging to the mosquito control operators (21 responses), Europe without France and Balkans (14), North Africa (12), Balkans (10), Turkey, South Caucasus and the Middle East (7), and unknown (1). A map of the origin of responses per country is provided in Supplementary file 4.

### Success rate of identification

The responders were significantly better at identifying females with 82% correct responses than larvae with 63% (*p* < 10^−5^ by Fisher’s exact test) (Table 1).

**Table 1:** Number of identifications (No. of IDs) at genus and species level for mosquito females (*n* = 26) and larvae (*n* = 12) within the total of 65 usable responses.

	No. of no IDs	No. of incorrect IDs	No. of correct IDs (%)
Genus			
Females	11/1690 (0.7%)	48/1690 (2.8%)	1631/1690 (96.5%)
Larvae	60/780 (7.7%)	23/780 (2.9%)	697/780 (89.4%)
Species			
Females	55/1690 (3.3%)	257/1690 (15.2%)	1378/1690 (81.5%)
Larvae	109/780 (14.0%)	183/780 (23.5%)	488/780 (62.6%)

**Figure 3:**
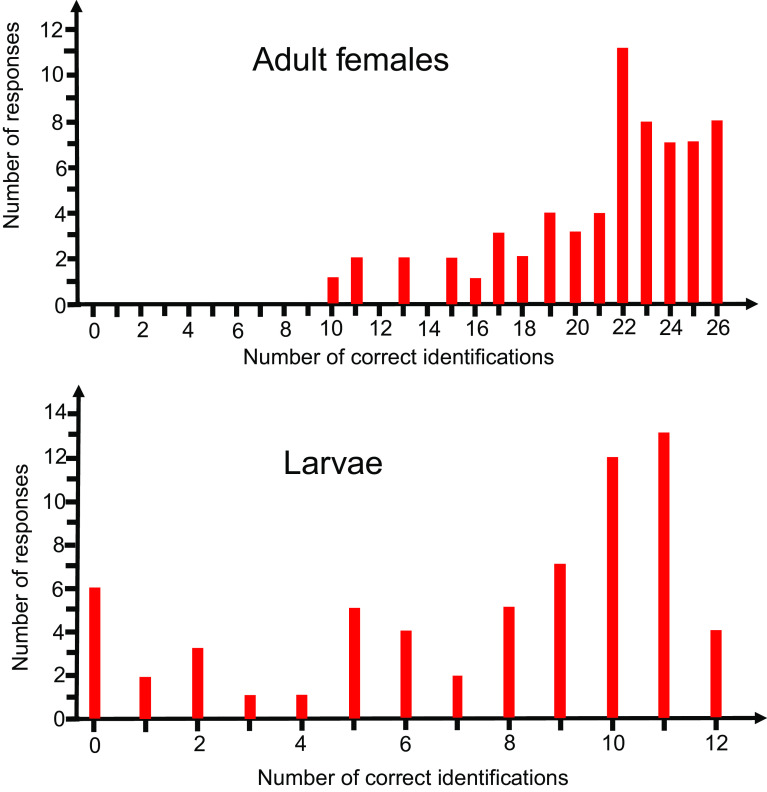
Distribution of the number of responses with regard to the number of correct identifications of mosquito species for 26 adult females and 12 larvae.

The range of correct IDs between species was very high (37%–97% for females and 18%–86% for larvae, Table 2). The range of correct IDs between responders was also very high (35%–100% for females and 0%–100% for larvae, Fig. 3).

**Table 2:** Number of identifications (No. of IDs) for 26 mosquito females and 12 larvae. Mosquito species are listed in decreasing percent correct responses. The category “miscellaneous” groups all IDs occurring only once.

Species	No. of no IDs (%/65)	No. of incorrect IDs (%/65)	No. of correct IDs (%/65)	Incorrect IDs (number of occurrences)
**Females**				
*Culiseta longiareolata*	1 (2%)	1 (2%)	63 (97%)	Miscellaneous (1)
*Aedes vexans*	0 (0%)	2 (3%)	63 (97%)	Miscellaneous (2)
*Aedes albopictus*	0 (0%)	2 (3%)	63 (97%)	Miscellaneous (2)
*Uranotaenia unguiculata*	3 (5%)	0 (0%)	62 (95%)	(0)
*Aedes caspius*	0 (0%)	3 (5%)	62 (95%)	Miscellaneous (3)
*Aedes aegypti*	0 (0%)	3 (5%)	62 (95%)	Miscellaneous (3)
*Culiseta annulata*	0 (0%)	4 (6%)	61 (94%)	Miscellaneous (4)
*Aedes vittatus*	2 (3%)	2 (3%)	61 (94%)	Miscellaneous (2)
*Coquillettidia richiardii*	1 (2%)	3 (5%)	61 (94%)	Miscellaneous (3)
*Orthopodomyia pulcripalpis*	4 (6%)	3 (5%)	58 (89%)	Miscellaneous (3)
*Culiseta subochrea*	2 (3%)	5 (8%)	58 (89%)	Miscellaneous (5)
*Aedes geniculatus*	2 (3%)	6 (9%)	57 (88%)	Miscellaneous (6)
*Culex theileri*	2 (3%)	6 (9%)	57 (88%)	Miscellaneous (6)
*Culex hortensis*	5 (8%)	3 (5%)	57 (88%)	Miscellaneous (3)
*Culex pipiens/torrentium*	2 (3%)	8 (12%)	55 (85%)	*Cx. martinii* (2), *Cx. perexiguus* (2), miscellaneous (4)
*Aedes japonicus*	1 (2%)	9 (14%)	55 (85%)	*Ae. koreicus* (5), miscellaneous (4)
*Aedes detritus/coluzzii*	2 (3%)	8 (12%)	55 (85%)	*Ae. leucomelas* (2), *Ae. flavescens* (2), miscellaneous (4)
*Anopheles plumbeus*	2 (3%)	11 (17%)	52 (80%)	*An. claviger* (8), miscellaneous (3)
*Anopheles sergentii*	1 (2%)	14 (22%)	50 (77%)	*An. superpictus* (5), *An. dthali* (3), *An. multicolor* (2), *An. cinereus* (2), miscellaneous (2)
*Anopheles claviger/petragnani*	1 (2%)	15 (23%)	49 (75%)	*An. sacharovi* (7), *An. marteri* (4), *An. algeriensis* (2), miscellaneous (2)
*Anopheles ziemanni*	2 (3%)	18 (28%)	45 (69%)	*An. hyrcanus* (12), *An. tenebrosus* (3), miscellaneous (3)
*Uranotaenia balfouri*	3 (5%)	19 (29%)	43 (66%)	*Ur. unguiculata* (17), miscellaneous (2)
*Anopheles dthali*	4 (6%)	22 (34%)	39 (60%)	*An. sergentii* (9), *An. superpictus* (6), *An. turkhudi* (3), *An. rhodesiensis* s.l. (2), miscellaneous (2)
*Culex poicilipes*	4 (6%)	24 (37%)	37 (57%)	*Cx. tritaeniorhynchus* (11), *Cx. vishnui* (3), *Cx. sitiens* (2), *Cx. thalassius* (2), miscellaneous (6)
*Aedes punctor*	7 (11%)	29 (45%)	29 (45%)	*Ae. hexodontus* (3), *Ae. cataphylla* (3), *Ae. pullatus* (3), *Ae. cinereus* (3), *Ae. intrudens* (2), miscellaneous (15)
*Culex tritaeniorhynchus*	4 (6%)	37 (57%)	24 (37%)	*Cx. mimeticus* (14), *Cx. sitiens* (4), *Cx. pipiens* (4), *Cx. vishnui* (4), *Cx. coronator* (2), *Cx. duttoni* (2), miscellaneous (7)
**Larvae**				
*Culiseta longiareolata*	7 (11%)	2 (3%)	56 (86%)	miscellaneous (2)
*Culex theileri*	8 (12%)	7 (11%)	50 (77%)	*Cx. pipiens* (2), miscellaneous (5)
*Aedes caspius*	9 (14%)	8 (12%)	48 (74%)	*Ae. vexans* (2), *Ae. detritus* (2), *Ae. punctor* (2), miscellaneous (2)
*Aedes aegypti*	9 (14%)	9 (14%)	47 (72%)	*Ae. albopictus* (5), miscellaneous (4)
*Culex pipiens*	8 (12%)	13 (20%)	44 (68%)	*Cx. torrentium* (3), *Cx. tritaeniorhynchus* (2), miscellaneous (7)
*Culex tritaeniorhynchus*	7 (11%)	14 (22%)	44 (68%)	*Cx. territans* (4), *Cx. pipiens* (2), *Cx. martinii* (2), *Ae. detritus* (2), miscellaneous (4)
*Uranotaenia balfouri*	10 (15%)	12 (18%)	43 (66%)	*Ur. unguiculata* (8), miscellaneous (4)
*Aedes vexans*	10 (15%)	15 (23%)	40 (62%)	*Ae. cinereus* (4), *Ae. cyprius* (3), miscellaneous (8)
*Aedes vittatus*	12 (18%)	17 (26%)	36 (55%)	*Ae. albopictus* (9), *Ae. aegypti* (4), miscellaneous (4)
*Aedes albopictus*	9 (14%)	18 (28%)	38 (59%)	*Ae. cretinus* (7), *Ae. geniculatus* (3), miscellaneous (8)
*Anopheles dthali*	10 (15%)	25 (38%)	30 (46%)	*An. sergentii* (14), *An. multicolor* (3), *An. gambiae* s.l. (3), *An. pulcherrimus* (2), miscellaneous (3)
*Anopheles ziemanni/coustani/tenebrosus*	10 (15%)	43 (66%)	12 (18%)	*An. maculipennis* s.l. (21), *An. hyrcanus* (13), *An. plumbeus* (2), *Ae. mariae* (2), miscellaneous (5)

For adult females, nine species obtained more than 90% correct IDs: *Culiseta longiareolata* (Macquart), *Aedes vexans* (Meigen), *Aedes albopictus* (Skuse), *Uranotaenia unguiculata* Edwards, *Aedes caspius* (Pallas), *Aedes aegypti* (Linnaeus), *Culiseta annulata* (Schrank), *Aedes vittatus* (Bigot) and *Coquillettidia richiardii* (Ficalbi). By contrast, four species obtained less than or equal to 60% correct IDs: *Anopheles dthali* Patton, *Culex poicilipes* (Theobald), *Aedes punctor* (Kirby) and *Culex tritaeniorhynchus* Giles. In case of larval ID, *Cs. longiareolata* was the most frequent correctly identified, while *Anopheles ziemanni* was the least frequent correctly identified species (Table 2).

Of note, three responders identified all the given materials 100% correctly (38 correct IDs out of 38 plates).

ID success was partly dependent on the regions of the responder. It was 85% in France, 77% in Europe without France and Balkans, 71% in Turkey, South Caucasus and the Middle East, 71% in North Africa and 57% in Balkans (percentages were not significantly different between the first three groups, but were different between the first group and the two latter groups, *p* < 0.05 by Mann–Whitney *U* test). An analysis of the ID success according to regions for both responders and species was hard to perform because most mosquito species exhibit a large distribution in the European and Mediterranean areas. However, in these areas, *Cx. tritaeniorhynchus* is restricted to the East of an imaginary line between Greece and Egypt, *An. dthali* and *Anopheles sergentii* (Theobald) are restricted to North Africa and the Middle East, and *Ae. vittatus* is restricted to Western Mediterranean Europe and Western North Africa [10]. As expected, comparisons of the ID success of these four species according to the presence/absence of the species in the country of the responder showed a higher success rate if the species is present (5 comparisons for the 4 species due to larva and female stages of *Cx. tritaeniorhynchus*) but none of these comparisons demonstrated a statistically significant difference (*p* always > 0.05 by Fisher’s exact test).

**Figure 4:**
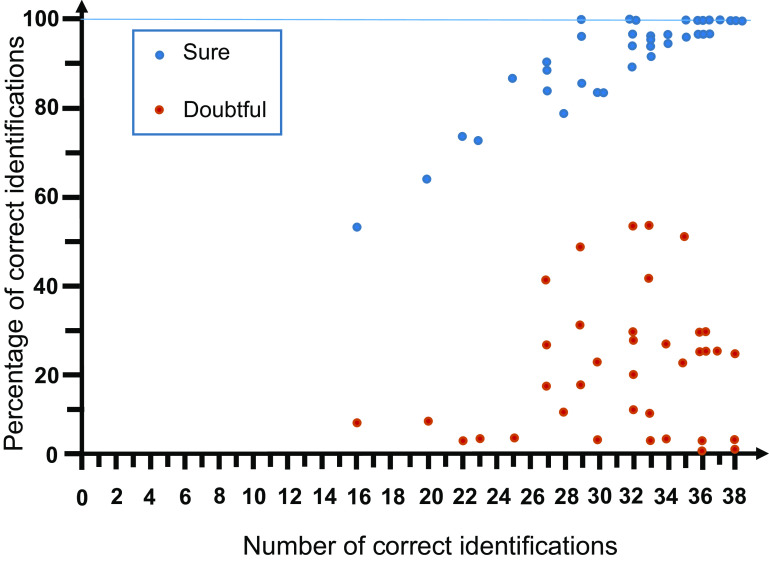
Distribution of the percentage of correct identifications estimated by the responder to be “sure” or “doubtful” with regard to the number of correct identifications for 38 mosquitoes (grouped for 26 females plus 12 larvae).

Of note, the organizers of the exercise kindly asked the responder to put the evaluation “sure” or “doubtful” for each ID. The proportion of IDs that were correct among the total IDs considered “sure” was 92% for females and 88% for larvae. These percentages were higher compared to the correct responses for the global dataset (as mentioned above, 82% and 63%, respectively; *p* < 10^−10^ by Fisher’s exact test, for both tests). Logically, the proportion of correct IDs out of total IDs estimated as “doubtful” was much lower, 65% for females and 54% for larvae. There was a strong correlation between IDs thought to be “sure” and the correct IDs (*r* = 0.89; *n* = 37). Although the lower correlation was demonstrated, this relationship was also verified between IDs thought to be “doubtful” and the correct IDs (*r* = 0.17, *n* = 37) (Fig. 4).

### Tools for identification

The most commonly used ID tools were MosKeyTool Version 2.2 by Günay et al. [3] (72% of responders), the dichotomous key from the publication by Becker et al. [1] (38%), the CD-ROM of Schaffner et al. [13] focusing on the mosquitoes of Europe (32%), the latter often associated with the CD-ROM of Brunhes et al. [2] focusing on the mosquitoes of North Africa, and the keys on the Walter Reed Biosystematics Unit (WRBU) website (https://www.wrbu.si.edu/vectorspecies/keys: 8%). Many other tools were used at a frequency ≤5%, including internet sources and old scientific literature (Supplementary file 5). Incidentally, one responder used, among several tools, a photographic key to adult female mosquito species of Canada and concluded logically but wrongly to mosquito species only distributed in the Americas.

The responders using MosKeyTool were significantly better in IDs (80% correct responses) than non-users (69%) (*p* = 0.024 by Mann-Whitney *U* test). The comparison between computer-aided tools (multi-criteria) versus dichotomous keys was not possible because of the small size of the sample (35 responders using exclusively computer-aided tools such as MosKeyTool [3] and the CD-ROM of Schaffner et al. [13] versus two responders using exclusively dichotomous keys such as the key by Becker et al. [1]).

Most of the responders (63%) used more than one ID tool. The percentage of correct responses was similar if respondents used a single tool (75%) or two or more tools (78%), without a significant difference (by Mann–Whitney *U* test).

Some IDs were performed just by personal expertise, without any tool, by 59% of responders (29/49 usable responses), with a mean number of 10% for female IDs and 4% for larva IDs and an extensive range between responders (0%–81% for female IDs and 0%–58% for larva IDs).

### Duration of the identifications

The mean and median duration of one ID (in min:s) was 10:48 and 8:20, respectively, for females; and 12:03 s and 9:58 s for larvae.

For females, the quickest responder (who had 100% correct ID) needed an average of 1:08 and the slowest 30:00 per ID (median: 18 s and 30 min, respectively), and 3:42 (the same responder with 100% correct ID) and 31:00 per ID for larvae, respectively (median: 4 min and 30 min, respectively).

For females, *Cs. longiareolata* had the quickest ID (5:12) and *Ae. punctor* the slowest one (21:30). For larva, *Cs. longiareolata* had the quickest ID (7:42) and *Ae. caspius* the slowest one (16:54).

## Discussion

To the best of our knowledge, this exercise is the second published EQA-like exercise concerning vector ID. The first one proposed by Jourdain et al. [4] also focused on female and larva mosquitoes. Although the two studies exhibited different study designs (Supplementary file 6); the ID success rates in the present study were higher for adults and similar for larvae (82% correct IDs for females and 63% for larvae; vs. 69% and 61%, respectively, in the abovementioned study by Jourdain et al.). When looking at the ID success rates, the average level of expertise may globally appear to be questionable if not insufficient. However, this must be modulated considering that most responders have seen several specimens for the first time, in particular when the species occurs in a region that is very distant from the geographical area of expertise of the responder.

The use of MosKeyTool was reported by 72% of responders. This indicates that MosKeyTool and other computer-aided illustrated ID tools which have a multi-criteria approach (i.e., non-dichotomous) are appreciated and respond to demand. More surprising is the continued use of CD-ROMs (Brunhes et al. [2], Schaffner et al. [13]) despite the fact that they have been integrated into MosKeyTool. This may be due to the obvious differences in the interfaces of these tools, the CD-ROM being perhaps perceived as more “user-friendly” than MosKeyTool/Xper2™ software.

The responders using MosKeyTool had higher correct ID rates than non-users (80% vs. 69%). This result supports and encourages the use of this tool in regional mosquito ID. However, as underlined several times here, the exercise design without randomisation in the assignment of a designated ID tool does not allow to conclude about the better quality of the tool compared to another one.

The high-quality photographs were much appreciated. Many participants commented that the specimens in the photos were in much better condition than specimens they have encountered before, which are often damaged when collected by mechanical traps (scales and even appendices are frequently lost) before the examination.

We observed a very low success rate in the ID of the larva of *An. ziemanni*, primarily confused with *An. maculipennis* s.l. (49% the incorrect IDs) and *An. hyrcanus* (30%). The fact that the specimen’s geographic origin was indicated in the template as Algeria added a difficulty because this species has not been previously found in Algeria but in all countries bordering Algeria [5].

This exercise underlined the potential but also the limits of morphological ID of mosquito specimens, at adult and larval stages, even using high-quality photos and state-of-the-art ID keys. It confirmed that regular mosquito identification practices and exchange of specimens within networks such MediLabSecure may help to improve the skill of the entomologists involved in mosquito surveillance activities [6]. However, morphological identification from actual specimens remains the gold standard method used so far to describe and identify mosquitoes. This demonstrates, once more, the need for an integrated taxonomy associating morphology with the complementary molecular approach, especially barcoding (PCR and gene sequencing) and MALDI-TOF mass spectrometry [8, 14] as far as possible.

Overall, the responders showed great enthusiasm while performing the exercise, and reported having enjoyed participating (Supplementary files 7 and 8). This suggests that there is potential to perform more exercises on a similar basis, possibly addressing specific questions, e.g., to compare ID keys, series of characters, optical equipment, or series of species.

The detailed description of the exercise here may be re-used in the future for any sort of comparable exercise (on mosquitoes or not), and the accessible images may be re-used for the development and internal validation of machine learning algorithms.

Finally, this exercise did make it possible to pinpoint some inaccuracies in MosKeyTool Version 2.2, such as morphological characters and distribution area, now adjusted in the recent upgraded Version 2.4.

## Supplementary data

The Supplementary materials of this article are available at https://www.parasite-journal.org/10.1051/parasite/2022045/olm *Supplementary file 1*: Read me first in English.
*Supplementary file 2*: Read me first in French.
*Supplementary file 3*: Template for identification results.
*Supplementary file 4*: Map of the countries.
*Supplementary file 5*: Tools used to identify mosquito species.
*Supplementary file 6*: Comparison of study design between the present study and Jourdain et al. (2018).
*Supplementary file 7*: Selection of comments in English.
*Supplementary file 8*: Selection of comments in French.

## Notes

**Cite this article as**: Rahola N, Günay F, Öztürk M, Alten B, Aqeehal HA, Saadawi WK, Shaibi T, Kavran M, Petrić D, Mitrović J, Pajovic I, Velo E, Kadriaj P, Rogozi E, Jani V, Manucharyan A, Paronyan L, Sawalha S, M’ghirbi Y, Bouattour A, Rhim A, Ouni A, Samy AM, Abozeid S, Sarih M, Assaid N, Arich S, Sokolovska N, Janceska E, Benallal KE, Haddad N, Zakhia R, Muja-Bajraktari N, Sherifi K, Arbaji M, Marić J, Santrac V, Dolidze N, Boussès P, Deblauwe I, Schaffner F & Robert V. 2022. Assessment of expertise in morphological identification of mosquito species (Diptera, Culicidae) using photomicrographs. Parasite **29**, 45.
