# Supplementary material for: Assessment of expertise in morphological identification of mosquito species (Diptera, Culicidae) using photomicrographs
Source: Parasite. 2022 Oct 6;29:45. doi: 10.1051/parasite/2022045 (PMC9536171; doi:10.1051/parasite/2022045)
Supplement: Supplementary file 1 — Read me first in English. Supplementary file 2: Read me first in French. Supplementary file 3: Template for identification results. Supplementary file 4: Map of the countries. Supplementary file 5: Tools used to identify mosquito species. Supplementary file 6: Comparison of study design between the present study and Jourdain et al. (2018). Supplementary file 7: Selection of comments in English. Supplementary file 8: Selection of comments in French. [file parasite-29-45-s1.pdf]

Supplementary data linked to the article:

Title: **Assessing the expertise for morphological identification of mosquito species (Diptera: Culicidae) through microphotographies**

Author: **Nil Rahola et al**

Published in: ***Parasite*** 2022

Supplementary file 1. Read me first in English (page 2)

Supplementary file 2. 'Read me first' in French (page 3)

Supplementary file 3. Template for identification results (page 4)

Supplementary file 4. Distribution of the countries (in red) with at least one responder (page 8)

Supplementary file 5. List of tools used to identify mosquito species (page 9)

Supplementary file 6. Comparison of study design between the present study and Jourdain et al (2018) (page 11)

Supplementary file 7. Selection of comments in English (page 12)

Supplementary file 8. 'Selection of comments' in French (page 13)

2022, 03 February

French version below

The European project MediLabSecure offers you the opportunity to evaluate your competence in the identification of mosquito species in the Europe-Mediterranean area.

In practice, this assessment involves three stages:

1) Download high resolution photo plates of 26 adult females and 12 four-instar larvae at https://mycore.core-cloud.net/index.php/s/kHSionPrXg0VRSi
This download (330 MB) is available now until 10 March 2022 included.
For your information, all the plates were made by Nil Rahola / IRD from mosquitoes kept in the ARIM (ARthropodes of Medical Interest) collection in Montpellier, France.

2) Proceed in your own way (dichotomous key, MosKeyTool, books...) to identify each species, either alone or with several colleagues. You have the possibility to zoom in very strongly to observe details on the areas of interest (setae, scales, teeth...). The photo of the leg shows a left hind leg unless otherwise indicated.
This identification being strictly morphological, the species of the same complex cannot of course be distinguished from one another, except in certain favourable cases by taking into account the origin of the photographed specimen (here Europe, North Africa, Middle East and Caucasus, i.e. the Western Palearctic ecozone).
You are invited to identify the 26 females and 12 larvae regardless of where they were collected.

3) Indicate the results of your identifications on the MS-Word document that is pre-filled. This document is joined with the plates; it indicates the geographical origin of the photographed specimen and gives some morphological info that may be useful for identification, especially for larvae.
This completed file (at least for the names of genera and species) must be returned as an attached file to vincent.robert@ird.fr by **Monday 14 March 2022** at the latest. This is the deadline for submitting your results in order to receive your final evaluation confidentially within 10 days (i.e. before 25 March).

Your result will be considered as a confidential document and its eventual further use will be exclusively statistical, and not nominative (see for example Jourdain et al, *Parasites & Vectors*, 2018, 11: 553. doi.org/10.1186/s13071-018-3127-7).

No prior registration is required. Participation is entirely free of charge and does not commit you to anything.

MediLabSecure

03 février 2022

Version anglaise ci-dessus

Le projet Européen MediLabSecure vous propose d'évaluer votre compétence en identification des espèces de moustiques de la zone Europe-Méditerranée.

En pratique, cette évaluation est à effectuer en 3 étapes :

1) Télécharger des planches de photos de haute définition, pour 26 adultes femelles et 12 larves de stade IV à https://mycore.core-cloud.net/index.php/s/kHSionPrXg0VRSi
   Ce téléchargement (330 Mo) est disponible dès maintenant jusqu'au 10 mars 2022 inclus.
   Pour information, toutes les planches ont été réalisées par Nil Rahola / IRD à partir de moustiques conservés dans la collection ARIM (ARthropodes d'Intérêt Médical) à Montpellier, France.

2) Procéder à votre manière (clé dichotomique, MosKeyTool, livres…) pour identifier chaque espèce, en procédant seul ou en vous groupant à plusieurs collègues. Vous avez la possibilité de zoomer très fortement pour observer des détails sur les zones d'intérêt (soies, écailles, dents…). Sauf indication contraire, la patte photographiée est une patte postérieure gauche.
   Cette identification étant strictement morphologique, les espèces d'un même complexe ne peuvent bien sûr pas être distinguées les unes des autres, sauf dans certains cas favorables par la prise en compte de la provenance du spécimen photographié (ici l'Europe, Afrique du Nord, Moyen-Orient et Caucase, soit l'écozone du paléarctique occidental).
   Vous êtes invité à identifier les 26 femelles et les 12 larves quelles que soient leur lieu de collecte.

3) Indiquer le résultat de vos identifications, en français ou en anglais, dans le fichier MS-Word qui vous est pré-rempli. Ce fichier est joint aux planches photos ; il indique l'origine géographique du spécimen photographié et donne des informations morphologiques utiles à l'identification, en particulier pour des larves.
   Ce fichier rempli (a minima pour les noms de genres et espèces) est à retourner en fichier attaché à vincent.robert@ird.fr au plus tard le **lundi 14 mars 2022**. C'est la date limite pour rendre vos résultats afin de recevoir confidentiellement sous 10 jours maximum (soit avant le 25 mars) votre évaluation finale.

Votre résultat sera considéré comme un document confidentiel et son éventuelle exploitation ultérieure sera exclusivement statistique, en aucun cas nominative (voir par exemple Jourdain et al, *Parasites & Vectors*, 2018, 11: 553. doi.org/10.1186/s13071-018-3127-7).

Aucune inscription préalable n'est requise. La participation est entièrement gratuite et ne vous engage à rien.

MediLabSecure Project -- WP Entomology -- Feb-March 2022

Response to the External Quality Assessment on identification (ID) of Euro-Mediterranean mosquito species (adults and larvae)

Responses to send as attached file by mail at vincent.robert@ird.fr before the 15th of March, 2022

Your name and E-mail address (mandatory):	

Code of photo plate	Country/area of collection	Hint	Genus name (required)	Species name (required)	What method of ID did you use? Please, see note under the table **(desired but not required)**	How long did you spend on your ID (approximately, in min) **(desired but not required)**	In your opinion, is your genus ID sure or doubtful? Please choose only from these 2 qualifiers **(desired but not required)**	In your opinion, is your species ID sure or doubtful? Please choose only from these 2 qualifiers **(desired but not required)**	Free commentary to justify/discuss your ID or explain why you estimate the ID impossible **(desired but not required)**
**MLS_A_01**	Central Europe	Big specimen							
**MLS_A_04**	Egypt								
**MLS_A_05**	Euro-Mediterranean								
**MLS_A_06**	Euro-Mediterranean								

**MLS_A_07**	Northern Italy	Left fore and mid legs are broken (top left photo)							
**MLS_A_08**	Morocco								
**MLS_A_09**	Georgia								
**MLS_A_10**	Algeria								
**MLS_A_11**	France								
**MLS_A_12**	Morocco								
**MLS_A_13**	Tunisia								
**MLS_A_14**	North Africa								
**MLS_A_15**	Turkey								
**MLS_A_21**	Egypt								
**MLS_A_23**	Western Mediterranean								
**MLS_A_25**	Balkan countries								
**MLS_A_26**	Western Europe								
**MLS_A_29**	Serbia								
**MLS_A_34**	Euro-Mediterranean								
**MLS_A_35**	Euro-Mediterranean								

**MLS_A_40**	UK								
**MLS_A_41**	Armenia								
**MLS_A_44**	Armenia								
**MLS_A_47**	France								
**MLS_A_49**	Spain								
**MLS_A_50**	Turkey	Fore leg available (on top of hind leg)							
**MLS_L_01**	Algeria								
**MLS_L_02**	Morocco	Antennae are finely spiculed ; head setae 5-C and 6-C are simple							
**MLS_L_03**	Euro-Mediterranean	Sub ventral setae of siphon 1-S measure 1/3 of the siphon diameter at point of insertion							
**MLS_L_04**	Egypt								
**MLS_L_05**	Morocco								

**MLS_L_06**	Balkans	Head setae 7-C have 2-3 branches							
**MLS_L_07**	North Africa, Middle-East	Head setae 3-C are simple ; thoracic setae 1-P are well developed and inserted on tubercles							
**MLS_L_08**	France								
**MLS_L_09**	Algeria								
**MLS_L_10**	Euro-Mediterranean								
**MLS_L_11**	Turkey								
**MLS_L_12**	Georgia								

Note : **What method of identification did you use?** For instance MosKeyTool, the key in Becker et al (2010), a home-made key, your own expertise without key, etc. Several methods are of course possible. If you identified 'by eye' because you know well the species, write ZERO.

Distribution of the countries (in red) with at least one responder.

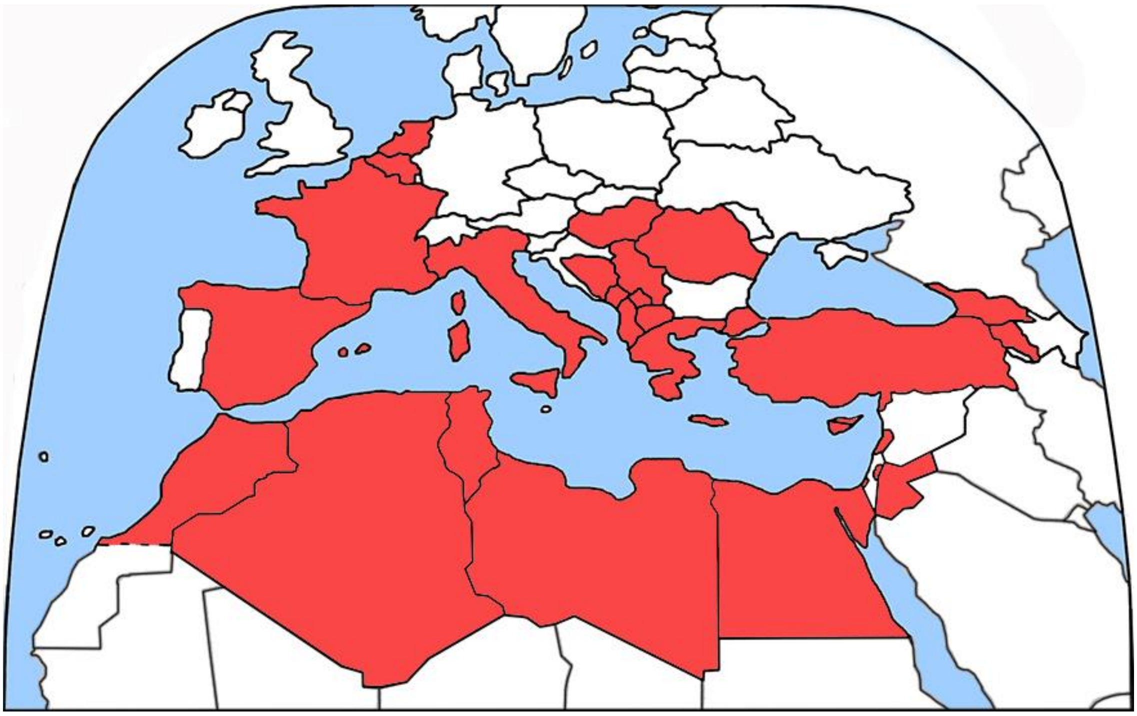

	number	%
MosKeyTool (Günay et al 2020)	43	72
Becker et al's book (2010)	23	38
CD-Rom Europe IRD (2001)	19	32
CD-Rom Afr Nord IRD (2000)	6	10
Personal key	6	10
WRBU website	5	8
Du Bose & Curtin (1965)	3	5
FactSheets MLS (MediLabSecure)	2	3
Harbach (1988)	2	3
Wilkerson et al's book (2021)	2	3
Coetzee (2020)	2	3
ECDC-EFSA (2021)	2	3
Rahola et al (2022	1	2
Harbach (1985)	1	2
Azari-Hamidian & Harbach (2009)	1	2
Severini et al (2009)	1	2
Severini et al (2022)	1	2
Glick (1992)	1	2
Petrić (2004)	1	2
Thielman & Hunter (2007)	1	2

Table. List of tools used for identifying mosquito species (n=60 responses)

Full references

Azari-Hamidian S, Harbach RE. 2009. Keys to the adult females and fourth-instar larvae of the mosquitoes of Iran (Diptera: Culicidae). Zootaxa 2078(1). https://doi.org/10.11646/zootaxa.2078.1.1

Becker N, Petrić D, Zgomba M, Boase C, Madon M, Dahl C, Kaiser A (2003, 2010, 2020) Mosquitoes and their control. Springer

Brunhes J, Rhaim A, Geoffroy B, Angel G, Hervy JP – *Les moustiques de l'Afrique méditerranéenne : logiciel d'identification et d'enseignement.* Paris (FRA); Tunis: IRD et IPT, 1 CD-ROM. (Didactiques). – Première version (2000) ; dernière mise à jour le 26/10/2011.

Coetzee M. 2020. Key to the females of Afrotropical *Anopheles* mosquitoes (Diptera: Culicidae). Malaria Journal 19(1), 70. doi: 10.1186/s12936-020-3144-9

Du Bose WP, Curtin TJ. 1965. Identification keys to the adult and larval mosquitoes of the Mediterranean area. J Med Entomol 1(4): 349-355.

Petrić D (2004) Culicidae Srbije i Crne Gore, kljue za zenke – rodovi. A poster. Laboratorija za medicinsku I veterinasku entomologiju, Poljoprivredni fakultet, UNS 2004

ECDC-EFSA. 2021. Reverse identification key for mosquito species. https://www.ecdc.europa.eu/en/all-topics-z/disease-vectors/surveillance-and-disease-data/reverse-identification-key-mosquito

FactSheets to identify 10 mosquito species of the Euro-Mediterranean area; Available at https://www.medilabsecure.com/resources_toolkit

Glick JI. 1992. Illustrated key to the female *Anopheles* of southwestern Asia and Egypt (Diptera: Culicidae). Mosquito Systematics 24(2): 125-153.

Günay F, Picard M, Robert V. 2020. MosKeyTool, an interactive identification key for mosquitoes of Euro-Mediterranean. Version 2.2. Available at http://medilabsecure.com/moskeytool. Last update: 23/06/2020

Harbach RE. 1985. Pictorial keys to the genera of mosquitoes, subgenera of *Culex* and the species of *Culex* (*Culex*) occurring in southwestern Asia and Egypt, with a note on the subgeneric placement of *Culex deserticola* (Diptera: Culicidae). Mosquito Systematics, 17: 83-107.

Harbach RE. 1988. The mosquitoes of the subgenus *Culex* in southwestern Asia and Egypt (Diptera: Culicidae). Contributions of the American Entomological Institute 24(1): 240 p.

Rahola N, Picard M, Petrić D, Robert V (2020) Simplified key to identify the 7 genera of modquitoes in Europe, North Africa and Middle-East, for larva and female. A poster MediLabSecure https://www.medilabsecure.com/resources_identification_key

Schaffner F, Angel G, Geoffroy B, Hervy JP, Rhaiem A, Brunhes J – Les moustiques d'Europe : logiciel d'identification et d'enseignement = The mosquitoes of Europe : an identification and training programme. Montpellier: IRD and EID, 1 CD ROM. (Didactiques). Première version (2001) ; dernière mise à jour le 31/08/2012.

Severini F, Toma L, di Luca M. 2022. Zanzare in Italia: raccolta, identificazione e conservazione delle specie più comuni. Roma: Istituto Superiore di Sanità. (Rapporti ISTISAN 22/3).

Severini F, Toma L, di Luca M, Romi R. 2009. Le zanzare Italiane: Generalità e identificazione degli adulti (Dipera, Culicidae). *Fragmenta Entomologica*, *41*(2), 213. https://doi.org/10.4081/FE.2009.92

Thielman AC, Hunter FF. 2007. A photographic key to adult female mosquito species of Canada (Diptera: Culicidae).Canadian Journal of Arthropod Identification No. 4 (December 2007) doi: 10.3752/cjai.2007.04

Wilkerson RC, Linton YM, Strickman D. 2021. Mosquitoes of the world. Johns Hopkins University Press (vol 1 & 2), 1308 p.

WRBU (Walter Read Biosystematics Unit) – Identification keys. https://www.wrbu.si.edu/vectorspecies/keys

Main differences in the study design between the two published studies aiming to identify mosquito species

	**Jourdain et al (2018)**	**The present study**
Object examined	specimens	plates of photographs
No. of adult mosqs to be examined per responder	7-8	28
Adult mosqs	males and females	females only
No. of larva mosqs to be examined per responder	4	12
Questions are identical for all responders	no	yes
Exercise open to non MediLabSecure laboratories	no	yes
Record of Sure/Doubtful identification	no	yes
Record of duration of each identification	no	yes
No. of usable responses	19	65

Jourdain F, Picard M, Sulesco T, Haddad N, Harrat Z, Sawalha SS, Günay F, Kanani K, Shaibi T, Akhramenko D, Sarih M, Velo E, Paronyan L, Pajovic I, Faraj C, Sikharulidze I, Putkaradze D, Maric J, Bosevska G, Janceska E, Bouattour A, Hamidi A, Sherifi K, Alten B, Petrić D, Robert V. 2018. Identification of mosquitoes (Diptera: Culicidae): an external quality assessment of medical entomology laboratories in the MediLabSecure Network. *Parasites and Vectors* 11(1), 553. doi: 10.1186/s13071-018-3127-7

Selection of comments (in English)

Some identifications require measurements of wings or appendices. This is not possible using the photographs in the absence of scale bars. Suggest adding scale bars where appropriate or possible.

I believe that our results would have been much better if we could have spent more time.

I didn't have time to look at the larvae, as I'm pretty busy with my PhD, and I have no expertise with larvae yet, so that was the reason to only identify the females.

I was on vacation the last couple of days, so, I have some times to work on the specimens. I think there are some windows for improving the MosKeyTool.

I'm relatively new to mosquito identification and there obviously is a lot of room for improvement but this was a great exercise, thanks a lot!

Prespiracular setae in *Culiseta annulata* are much better visible than under the stereo microscope.

Congratulations on this initiative, the photos are spectacular. It's been so much fun.

I'm so sorry about my bad results. Moreover, at the last minute I changed three right results to wrong ones.

I am really pleased that most of my answers are correct. Nevertheless, I am pretty angry at myself for having mistaken one of the most common species, *Ae. albopictus*! Thank you once again for the opportunity to test my competence.

What a great MediLabSecure initiative to organize a test for ID skills, thanks a lot!

Thank you for this nice test. Let's hope that for the future tests we will be able to meet face-to-face with real specimens.

It was interesting doing this kind of exercises, we learned a lot.

I had a lot of fun testing my knowledge in mosquito identification and I will most certainly focus more on improving my knowledge in larval stage in the future. If you are planning similar tests in the future I would love to participate again.

The photos are really nice and the specimens perfect. So if you use one or two keys you should find the species. I prefer to check myself with different keys. It is always nice to learn to identify new mosquitoes.

I didn't work with larvae, so I don't have the knowledge to identify them. I hope to learn in the future.

I did not perform larval samples related to the lack of basic knowledge about the larval morphology. I understand to needing learn larval morphology.

It has been very interesting and we enjoyed it a lot! We hope we have done all with no mistakes and we will be waiting to know our score! We hope this will help you to continue working in this field.

Congratulations on this initiative, the photos are spectacular. It's been so much fun.

This quiz was fun and very educational thanks to your feedback. Please tell Nil that he's done an incredible job.

What a great initiative to organize a test for ID skills, thanks a lot! Many of the North African species are new for me, we never see those here of course. But great fun to try. And not to forget: many thanks & compliments to the photographer who made all those excellent pictures. Outstanding!

I would like to thank the MediLabSecure Project team for this kind of exercise, so useful to simultaneously evaluate and ameliorate our knowledge in mosquito's taxonomy.

Some of the specimens we were identifying for the first time, but we consider this experience very helpful. The description of the specimens now are clearer for us and the plates of photos you will provide will be very useful.

It was really a pleasure and improvement skills to the identification, especially for larvae.

About larvae, my experience is very limited with their identification. Usually, when we catch larvae from the field, we let them to rearing and we make species identification on the adult stage because is easiest.

Sélection de commentaires (en Français)

C'est un super outil de formation!

C’est vraiment super bien fait ! Après, ce n’est pas toujours facile de voir la couleur exacte des écailles selon la luminosité et l'éclairage peut influencer la détermination.

Merci pour avoir organisé cet exercice qui nous obligé de nous remettre dans le bain des identifications. Je dois aussi reconnaitre que les plaquettes des photos étaient d’une grande qualité. Bravo à Nil. On s’est débrouillé avec mon collègue qui a fait les identifications des adultes et moi j’ai fait les larves.

Je vous remercie pour cette opportunité d'évaluation de mes compétences en identification proposée par le projet MediLabSecure.

Merci pour ton retour qui me rassurent globalement. Sauf pour la larve d'*Aedes albopictus*, je suis impardonnable, il est grand temps que je retourne un peu plus sur le terrain !!!

Heureusement que le pays / région est indiqué(e) !

J’ai vraiment été ravi d’apporter ma petite contribution à un super projet. J’espère qu’un Atlas verra le jour car on en a vraiment besoin.

J'ai commencé avec les deux clés : CD-ROM-IRD et MosKeyTool. Mais je me suis rendu compte que je n'aboutissais pas aux mêmes résultats, alors j'ai travaillé uniquement avec CD-ROM. Les photos sont très belles. Au final ça confirme mon ressenti, je déteste les imagos de *Culex* et les larves d’*Anopheles*.

C’est vraiment super bien fait ! Après, ce n’est pas toujours facile de voir la couleur exacte des écailles selon la luminosité … comme en vrai.

Etant débutant en identification de moustiques ce type d’exercice est précieux. Par manque de temps je me suis concentré sur MosKeyTool, mais je vais approfondir la clé de Becker.

Je m'interroge concernant les droits d'utilisation des images.

Nous avons travaillé parfois en petit groupe, parfois seul et avons regroupé nos résultats en un seul fichier.

C’était vraiment très sympa de faire de l’ID sur des photos comme celles-ci !! Du luxe !!

L’exercice était très plaisant à faire ; ma collègue trouvait même cela addictif à la fin.

La qualité des photos a été appréciée. Cela changeait des signalements que nous recevons de particuliers avec des photos pixélisées ou des insectes écrasés.

Afin de m’accoutumer à la clé et améliorer mes connaissances générales sur le sujet, dans un deuxième temps j’ai systématiquement tout repassé au crible avec MosKeyTool. Pour quelques espèces, l’identification a pu prendre bien plus de 30 min car il a été nécessaire de revenir dessus plusieurs fois et j’ai aussi profité de ce travail pour décortiquer au maximum les identifications. Certains critères dans MosKeyTool font appel à des mesures mais il n’y avait pas d’échelle pour faire les comparaisons.

Il faut vraiment jongler avec MKT et d’autres clés (Becker et moustiques d’Europe).

Concernant la dernière larve MLS_L_10, j’ai douté car bien que tous les critères m’amenaient à *Anopheles ziemanni*, l’espèce est notée absente en Algérie dans MosKeyTool.

Cet exercice m’a permis de me remettre dans les identifications et même de découvrir des critères pour les larves que j’identifie rarement et sur lesquelles je pense être moins bon.

C'est un super outil de formation!
